# Circulating total 25(OH)D and calculated free 25(OH)D in professional academy footballers at a northerly latitude in the UK

**DOI:** 10.5114/biolsport.2024.131822

**Published:** 2023-11-10

**Authors:** Simon D. Bowles, Subhashis Basu, Mayur K. Ranchordas, Trevor Simper, Anthony Lynn

**Affiliations:** 1 Food Group, Sheffield Business School, Sheffield Hallam University, Sheffield, UK; 2 Sheffield Teaching Hospitals NHS Trust, Sheffield, UK; 3 Academy of Sport & Physical Activity, Faculty of Health & Wellbeing, Sheffield Hallam University, Sheffield, UK; 4 Advanced Wellbeing Research Centre, Sheffield Hallam University, UK

**Keywords:** Total 25(OH)D, Free 25(OH)D, Free 1, 25(OH)2D, Footballers

## Abstract

There is limited data on the vitamin D status of UK-based professional academy footballers. Therefore, the objective of this study was to report total 25(OH)D, free 25(OH)D and free 1, 25(OH)_2_D at the end of the winter (March) and summer periods (October) in a cohort (n = 27) of professional academy footballers in northern England. Blood samples were collected to measure total 25(OH)D, parathyroid hormone, vitamin D binding protein, albumin and calcium. Free 25(OH)D and 1, 25(OH)_2_D were calculated. Dietary vitamin D intake and retrospective summer sunlight exposure were also collected. At the end of winter, 2/27 (7.4%) players were vitamin D deficient (25(OH)D < 30 nmol/l) and 11/27 (40.7%) were insufficient (25(OH)D > 30 nmol/l < 50 nmol/l). By the end of summer, none were deficient but 3/14 (21.4%) were still insufficient. Median total 25(OH)D (82.2 nmol/l [IQR: 50.3–90.2] vs. 54.2 nmol/l [IQR: 36.8–71.9]; P = .02), free 25(OH)D (25.8 pmol/l [IQR: 15.1–33.1] vs. 13.2 pmol/l [IQR: 9.0–14.9]; P = .005) and free 1, 25(OH)_2_D (389 fmol/l [IQR: 209–594] vs. 212 fmol/l [IQR: 108–278]; P = .034) were significantly higher at the end of summer than the end of winter. At the end of winter, free 25(OH)D was lower (P = .003) in those vitamin D insufficient (8.8 pmol/l [IQR: 5.5–11.8]) vs. sufficient (13.7 pmol/l [IQR: 12.0–17.0]). There was a high prevalence of vitamin D insufficiency at the end of the winter. Free 25(OH)D was also lower at the winter timepoint and in players that were insufficient vs. sufficient.

## INTRODUCTION

In addition to its involvement in calcium and phosphate homeostasis, vitamin D has been associated with important roles that are relevant to athletes [1–5]. Vitamin D status in footballers has been shown to correlate with measures of physical performance, including VO_2max_, sprint times and vertical jump height [1]. Vitamin D deficiency and insufficiency may also impair immune function in athletes [4] and influence injury incidence [5].

A meta-analysis of 23 studies involving 2, 313 athletes showed that 56% of athletes across different geographical locations were vitamin D insufficient. The prevalence of vitamin D insufficiency was highest in the winter and spring seasons and was significantly higher in the UK than other geographical locations, including Spain, France, Australia, and the USA [6]. Vitamin D status is therefore of particular interest to UK sportspersons, but currently there is limited information in this area in UK-based footballers. The two existing studies from the UK to date have been carried out on professional players [2, 7]. One of these studies found that 55% of footballers had a circulating 25(OH)D < 50 nmol/l [2] during the winter (November-January) months, but this data was based on a sample of 11. A brief communication also reported 65% of 20 Premier League footballers were vitamin D insufficient in the winter [7]. To our knowledge no studies have reported vitamin D status in UK-based professional academy footballers.

Vitamin D status is currently assessed by measuring circulating total 25-hydroxyvitamin D (25(OH)D) because it reflects contributions from cutaneous synthesis and from the diet and has a relatively long circulating half-life of 13–18 days [8]. The gold-standard method for measuring total 25(OH)D concentration is liquid chromatography mass-spectrometry (LC-MS/MS) [9]. So far, no study has reported vitamin D status in UK footballers using this method and only a few studies have used this method in other European countries [10]. Whilst one previous study in the UK has reported seasonal variations in total 25(OH)D level in UK footballers (in August and December) [7], as far as we are aware, no studies have been conducted at the optimal time point to capture the winter vitamin D nadir (March).

Existing studies in UK footballers have also only reported total 25(OH)D to assess vitamin D status. There has been debate as to whether this is the most appropriate biomarker to use [11]. More specifically, around 85% – 90% of total 25(OH)D and 1, 25(OH)_2_D in circulation is bound to vitamin D binding protein (VDBP) and around 10% – 15% is bound to albumin. Unbound or free 25(OH)D accounts for < 1% of total circulating vitamin D [11] but the free hormone hypothesis suggests that only hormones that are liberated from their carrier proteins exert a physiological effect [12]. In healthy adults, a stronger association has been shown between free 25(OH)D and bone mineral density [13] and free 25(OH)D and parathyroid hormone (PTH) [14] than total concentrations. Therefore, free 25(OH)D concentrations may be an important metabolite to consider in athletes.

To our knowledge, no previous studies have assessed total 25(OH)D in UK-based professional academy football players at the optimal seasonal timepoints using the gold-standard approach and reported free vitamin D metabolites. Therefore, the purpose of this study was to examine these in a cohort of UK-based academy players at a professional football club in northern England.

## MATERIALS AND METHODS

### Participants

A convenience sample of 27 male professional footballers (aged 16–21 years) from a football academy situated at a northerly latitude in the UK (53^o^ North) were recruited to the study. The study was approved by the Institutional Ethics Committee (SBS16/17136). Exclusion criteria were injury or illness within 6 weeks prior to measurement of blood biochemistry and/or unwillingness to complete study measurements (e.g., phobia of venipuncture). All data collection was performed in accordance with the ethical standards of the Helsinki Declaration and all participants provided written informed consent.

### Study design

This is a cross-sectional, observational study to determine the vitamin D status of this cohort of footballers at the end of winter and the end of summer. Measurements were taken in March, when vitamin D status is expected to be at its annual nadir, and the beginning of October when there has been opportunity for endogenous synthesis of vitamin D over the summer months and, therefore, vitamin D levels are likely to be at their peak [15].

At the end of winter timepoint, blood samples were collected to measure serum total 25(OH)D, 1, 25(OH)_2_D, parathyroid hormone (PTH), vitamin D binding protein (VDBP), albumin, calcium, creatinine and phosphate. Free 25(OH)D and free 1, 25(OH)_2_D were calculated. Dietary vitamin D intake was collected by an estimated fourday dietary record and retrospective summer sunlight exposure was collected by questionnaire. At the end of summer timepoint, a further blood sample was collected from each participant for repeated determination of vitamin D status and related biochemistry.

### Study measurements

#### Anthropometric measurements

Height was measured in metres to the nearest 0.1 centimetre using a stadiometer and body mass to the nearest 0.1 kilogram using an electronic balance scale. Body mass index (BMI) was calculated using Quetelet’s index.

### Sample handling

Samples were collected into serum separating tubes (SST). Blood in SST tubes was left to clot for 30 minutes at room temperature and centrifuged at 3000 g for 10 minutes. The serum was aliquoted and stored at -80^o^C until analysis.

### Total serum 25(OH)D

Total 25(OH)D_2_ and 25(OH)D_3_ were measured by liquid chromatography with tandem mass spectrometry (LC-MS/MS) which is the method used by the National Institute of Standards and Technology [16]. Measurements were carried out at the laboratory of the Institute of Human Development (University of Manchester, UK). This laboratory participates in the Vitamin D External Quality Assessment Scheme, and the assay was calibrated against the National Institute of Standards and Technology standard. Samples (200 *μ*l) and a deuterated internal standard (d_6_-25(OH)D were prepared using 100 *μ*l methanol:isopropanol (80:20) and then extracted with 1 ml of hexane. This extracted 25(OH)D was blown down, reconstituted in 150 *μ*l of 66% methanol and injected onto a waters phenyl column attached to the mass spectrometer. The extract was eluted with an isocratic gradient over 5 minutes. Analysis was carried out in positive ion mode using the transitions m/z 401 > 159 for 25(OH)D and Mm/z 407 > 159 for d_6_-25(OH)D.

In this study, vitamin D deficiency (total 25(OH)D < 30nmol/l) and insufficiency (total 25(OH)D between 30 nmol/l and 50 nmol/l) was defined using the Institute of Medicine classifications [8] and these thresholds are widely used by UK healthcare practitioners.

### Other biochemistry

Vitamin 1, 25(OH)_2_D was measured by chemiluminescence immuno assay (CLIA) after an extraction step on the IDS-iSYS (Immunodiagnostic Systems, Boldon, UK) at the Bone Biochemistry Laboratory (University of Sheffield, UK). The inter-assay CV was 6.0%. Intact PTH was measured by an automated sandwich CLIA on the IDS-iSYS (Immunodiagnostic Systems, Boldon, UK) at the Bone Biochemistry Laboratory (University of Sheffield, UK). The manufacturers interassay CV is < 2.0%. Serum albumin, calcium, creatinine, and phosphate were measured using an automated colorimetric assay on the Cobas c701 (Roche Diagnostics, Mannheim, Germany) in the Chemical Chemistry laboratory (Sheffield Teaching Hospitals, UK). The inter assay precision was < 2.0% for all tests. Vitamin D binding protein (VDBP) was measured using a polyclonal non-competitive two-site enzyme-linked Sandwich immunoassay (Genways, San Diego, USA). The inter- and intra- assay CV were 3.9% and 3.3%, respectively.

### Calculated free 25(OH)D and free 1, 25(OH)_2_D

Free 25(OH)D and 1, 25(OH)_2_D was calculated using the concentrations of albumin and VDBP and their respective binding affinities for 25(OH)D.

The formula used for free 25(OH)D was [17]:


$$\text{Free }25(\text{OH})\text{D}=\frac{\text{Total }25(\text{OH})\text{D}}{1+(6\times 10^{5}\times \text{albumin})+7\times 100^{8}\times \text{VDBP}}$$

The formula used for 1, 25(OH)_2_D was [18]:


$$\begin{array}{l} \text{Free }1,\;25{\left(\text{OH}\right)}_{2}\text{D}=\\ \frac{\text{Total }1,\;25{\left(\text{OH}\right)}_{2}\text{D}}{1+(5.4\times 10^{4}\text{M}-1\times \text{albumin})+(3.7\times 10^{7}\text{M}-1\times \text{VDBP})} \end{array}$$

### Dietary vitamin D and sunlight exposure assessment

Participants were asked to complete an estimated 4-day diet diary to assess dietary intake of vitamin D at the end of winter timepoint. Data was collected on two training days, one match day and one rest day. After completion, a trained nutritionist interviewed the participants to establish the accuracy of the completed diaries. Dietary vitamin D intake was calculated using Nutritics (Dublin, Ireland).

Habitual sunlight exposure was estimated at baseline using a retrospective sunlight exposure questionnaire that was adapted from a previous study [19]. Despite known difficulties in quantifying sun exposure due to many influencing factors [20], data derived from questionnaires has been shown to be correlated with vitamin D status [21].

For each month of the year participants were asked to score how often they were usually outside and exposed to the sun. A score of 3 for ‘often’, ‘2’ for occasionally, and ‘1’ for seldom. The score given was multiplied by the total body areas exposed in each month by using the percentages below from the ‘rule of nine’ to estimate the surface area of the skin exposed to sunlight. The ‘rule of nines’ is a tool used by medical practitioners to assess the total body surface area involved in burns patients. It has previously been used in vitamin D research to estimate the surface area of the skin exposed to sunlight [22]:

– Head = 9% (front and back together)
– Both arms = 9% (L) + 9% (R)
– Both legs = 18% (L) + 18% (R)
– Torso = 18% (front) + 18% (back)
– Groin = 1%

For example, a participant ticking ‘occasionally’, and ‘head’ and ‘arms’ would get a score for that month of (2*(0. 09 + 0.09 + 0.09)) = 0.54. By using this scoring system, a score for ‘summer sunlight exposure’ was derived by calculating the sum of the scores for months where endogenous vitamin D synthesis can occur in the UK (April to September).

### Statistical analysis

Statistical analyses were performed using the Statistical Analysis Package for Social Sciences (SPSS V.26 for Windows, Chicago, Illinois, USA).

Each variable is presented as median and inter-quartile range (IQR) due to the non-normal distributions. Each variable was tested for normality with a Shapiro-Wilk test. Normality was assumed if Shapiro-Wilk test was not statistically significant (*P* > .05) but no paired- or between-participant variables met the assumptions of parametric testing. Therefore, non-parametric Mann-Whitney U test was used to assess for differences between the groups. For paired comparisons of variables, a non-parametric Wilcoxon test was used. A Pearson’s correlation coefficient (where Shapiro-Wilk was P > .05) or Spearman’s Rho (where Shapiro-Wilk was P < .05) were used to determine significant relationships between variables. For all tests a P < 0.05 was set as the critical level of significance.

## RESULTS

Blood samples were obtained in March from all 27 participants. Thirteen of the participants had left the Academy by October, so samples were captured from the remaining 14 players. Completed diet diaries and sunlight exposure questionnaires were obtained from twenty participants. In all blood samples, vitamin 25(OH)D_2_ was undetectable and so we have not reported concentrations of this metabolite. The participant characteristics at the start of the study at the end of winter timepoint are shown in Table 1.

**TABLE 1:** Participant characteristics (n = 27) from the end of winter (March) timepoint.

Variables	Median	IQR
Age (years)	18	17–18
Height (cm)	179.4	174.1–185.3
Body mass (kg)	76.2	67.5–81.6
BMI (kg/m^2^)	23.2	22.3–24.0
Total 25(OH)D (nmol/l)	50.2	40.4–61.8
Total 1, 25(OH)_2_D (pmol/l)	25.5	15.1–33.4
Calculated free 25(OH)D (pmol/l)	12.8	8.8–14.9
Calculated free 1, 25(OH)_2_D (fmol/l)	242	174–349
Parathyroid Hormone (pg/ml)	31.7	21.8–42.0
Serum Phosphate (mmol/l)	1.19	1.14–1.28
Serum Creatinine (*μ*mol/l)	94	87–103
eGFR (ml/min/1.73m^2^)	88	79–90
Serum Calcium (mmol/l)	2.42	2.37–2.48
Adjusted Serum Calcium (mmol/l)	2.19	2.15–2.23
Albumin (g/l)	52	51–54
Vitamin D Binding Protein (*μ*g/ml)	322	273–391
Total Alkaline Phosphatase (IU/l)	101	78–124

### Vitamin D metabolites

**FIG. 1:**
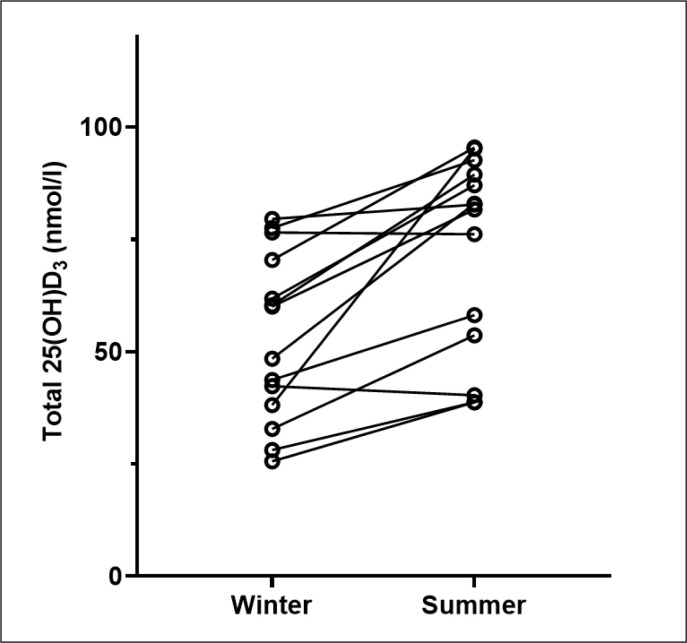
Change in median total 25(OH)D status from the end of winter (n=14) the to end of summer (n = 14). Circles represent individual participant values

At the end of winter, 2/27 (7.4%) players were vitamin D deficient (25(OH)D < 30nmol/l), 11/27 (40.7%) were insufficient (25(OH)D > 30nmol/l < 50 nmol/l) and 14/27 (51.9%) were sufficient. By the end of summer, none were deficient, 3/14 (21.4%) were insufficient and 11/14 (78.6%) were sufficient. Analysis of a subset where we had March and October samples was completed (n = 14). Total 25(OH)D concentration was lower at the end of winter (median: 54.2 nmol/l [IQR: 36.8–71.9]) compared to the end of summer (median: 82.2 nmol/l [IQR: 50.3–90.2]) and this difference was significant (Z = -3.107, P = .02) (Figure 1).

Calculated free 25(OH)D and calculated free 1, 25(OH)_2_D were significantly lower at the end of winter than the end of summer. PTH and VDBP were significantly higher at the end of winter than the end of summer but there was no significant difference in adjusted serum calcium or albumin between the two timepoints (Table 2).

**TABLE 2:** End of winter (March) and end of summer (October) values for PTH, adjusted serum calcium, VDBP, albumin and other vitamin D metabolites.

Variables	March (n = 14)		October (n = 14)		P-value

	Median	IQR	Median	IQR	
PTH (pg/ml)	33.2	25.1–47.0	21.7	17.1–24.5	.004
Adjusted Serum Calcium (mmol/l)	2.20	2.13–2.26	2.16	2.10–2.19	.345
Total 1, 25(OH)_2_D (pmol/l)	15.6	12.9–32.8	25.1	15.7–36.5	.140
Calculated free 25(OH)D (pmol/l)	13.2	9.0–14.9	25.8	15.1–33.1	.005
Calculated free 1, 25(OH)_2_D (fmol)	212	108–278	389	209–594	.034
VDBP (*μ*g/ml)	338	278–407	204	179–232	.005
Albumin (g/l)	52	50–54	51	49–53	.555

At the end of winter timepoint, there were no statistically significant differences in total 1, 25(OH)_2_D, calculated free 1, 25(OH)_2_D, PTH, adjusted serum calcium, VDBP or albumin between players who were insufficient (total 25(OH)D < 50nmol/l) and sufficient (total 25(OH)D > 50nmol/l). However, calculated free 25(OH)D was significantly higher in those who were sufficient compared to those who were insufficient (Table 3).

**TABLE 3:** PTH, adjusted serum calcium, VDBP, albumin and other vitamin D metabolites in players who were insufficient (total 25(OH)D < 50 nmol/l) vs. players who were sufficient (total 25(OH)D >50 nmol/l) at the end of winter (March).

Variables	Deficient/Insufficient (n = 13)		Sufficient (n = 14)		P-value

	Median	IQR	Median	IQR	
PTH (pg/ml)	29.2	22.2–39.7	33.2	17.3–48.5	.756
Adjusted Serum Calcium (mmol/l)	2.2	2.16–2.22	2.19	2.15–2.24	.805
1, 25(OH)_2_D (pmol/l)	24.3	13.2–29.8	27.6	17.3–27.6	.225
Calculated free 25(OH)D (pmol/l)	8.8	5.5-11.8	13.7	12.9-17.0	.003
Calculated free 1, 25(OH)2D (fmol/l)	212	154-334	242	212-357	.432
VDBP (*μ*g/ml)	321	236-384	323	273-393	.667
Albumin (g/l)	51	49-54	53	51-54	.128

### Correlations between total 25(OH)D, free 25(OH)D and PTH

There was no relationship between total 25(OH)D and PTH at the end of winter (r = -.033, P = .870) or end of summer timepoints (r = -.415, P = .158). There was also no relationship between free 25(OH)D and PTH at the end of winter (r = -.033, P = .870) or end of summer timepoints (r = -.033, P = .870).

**FIG. 2:**
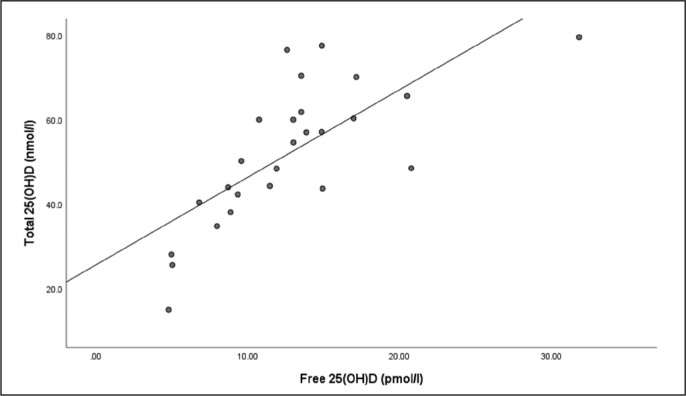
The relationship between total 25(OH)D and free 25(OH)D at the end of winter timepoint.

**FIG. 3:**
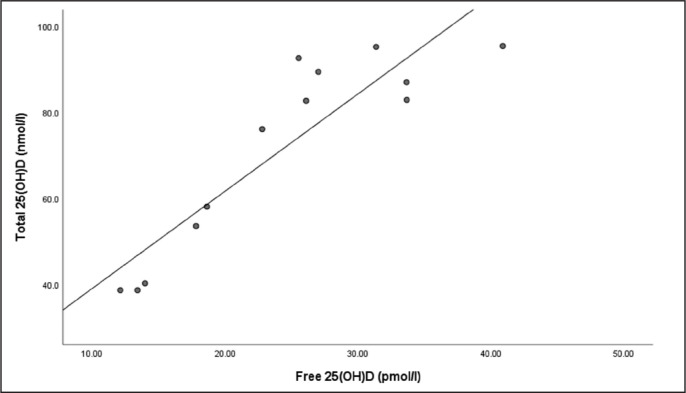
The relationship between Total 25(OH)D and free 25(OH)D at the end of summer timepoint.

There were strong relationships between total 25(OH)D and free 25(OH)D at the end of winter (r = .766, P = < .001) (Figure 2) and end of summer timepoints (r = .883, P < .001) (Figure 3).

### Dietary vitamin D & sunlight exposure

Overall median dietary vitamin D intake was 2.8 *μ*g/day (IQR: 1.6–6.3). Median dietary vitamin D intake in players with sufficient 25(OH)D concentrations was 3.7 *μ*g/day (IQR: 2.8–8.8), whereas it was 1.6 *μ*g/day (IQR: 1.3–3.3) in players with insufficient 25(OH)D concentrations. This difference was statistically significant (U = 15.0, n_1_ = 11, n_2_ = 9, P = .007).

The median summer sunlight exposure score in players that were sufficient in 25(OH)D was 10.18 (IQR: 8.74–11.62) and was 9.93 (IQR: 8.12–11.42) in those that were insufficient, but this difference was not significant (U = 46.5, n_1_ = 11, n_2_ = 9, P = .824).

## DISCUSSION

Our study has identified a high prevalence of total 25(OH)D_3_ insufficiency in professional academy football players at the end of winter (7.4% vitamin D deficient and 40.7% insufficient). To our knowledge, no other study has reported total 25(OH)D concentrations in professional academy footballers in the UK, but our data are congruent with findings from other studies of academy and professional level football players across Europe, which have demonstrated winter insufficiency rates of up to 75% [2, 7, 23–25].

The fall in total 25(OH)D_3_ concentration from the end of summer to the end of winter in professional football players has previously been established [7] and this reflects that of the general UK population [15]. Findings from our study also show that total 25(OH)D was higher at the end of summer (median: 82.2 nmol/l) compared to the end of winter (median: 54.2 nmol/l). However, even after the summer period around one fifth of players tested were still insufficient (21.4%). This prevalence of insufficiency is much higher in comparison to other studies of elite football players in Europe that have reported summer period total 25(OH)D concentrations [7, 23, 26] but these studies were carried out on professional first team, rather than academy level players. This is interesting and, taken with the vitamin D insufficiency rates from the winter period, may highlight a lack of nutrition related advice provided to academy compared to professional players and/or poor compliance with nutrition-related advice in academy level players. These findings are also important when we consider that adequate vitamin D status may be important for optimal performance [1–3, 10, 27] and injury prevention [5, 28], particularly in professional academy players who typically have greater training loads in comparison to the first team [29].

Total 1, 25(OH)_2_D was not different between the two timepoints. This is not surprising due to tight homeostatic control of this metabolite [30]. However, our results show that PTH concentration was lower in academy players at the end of summer compared to the end of winter, although it was within the normal range (10–65 ng/ml) at both time points. A study in professional Polish football players has also shown lower concentrations of total 25(OH)D (62.4 ± 24.8 nmol/l vs. 77.1 ± 22.6 nmol/l, P = 0.0003) and higher PTH (25.37 ± 7.95 pg/ml vs. 21.37 ± 6.88 pg/ml, P = 0.0062) at the end of winter compared to the end of summer [31]. PTH influences bone turnover and therefore future studies should consider any detrimental effects of low 25(OH)D on bone health outcomes in young professional footballers. However, despite the difference in PTH in our study, there were no differences in serum calcium concentration between the two timepoints. There was also no relationship between PTH and total 25(OH)D or free 25(OH)D at either timepoint. Other studies in footballers have also failed to detect a significant association between total 25(OH)D and PTH [26, 32]. However, this is likely due to the small sample sizes in these studies and the relatively weak inverse association previously reported between total 25(OH)D and PTH at a population level [33]. To our knowledge no study has previously reported the association between free 25(OH)D and PTH in footballers but a significant inverse association has been demonstrated in non-athlete populations [34].

Due to recent interest in free vitamin D metabolites, we also report free 25(OH)D and free 1, 25(OH)_2_D. Free 25(OH)D was similar to concentrations reported elsewhere in healthy people [12, 35]. Like total 25(OH)D, we found a lower free 25(OH)D at the end of winter compared to the end of summer. We also found a strong correlation between total 25(OH)D and free 25(OH)D at both study timepoints. This is congruent with literature from other population subgroups where free 25(OH)D has been shown to be highly correlated with total 25(OH)D [14, 34, 36, 37]. Some studies have shown that free 25(OH)D might be better correlated with health and biochemical outcomes than total 25(OH)D, including BMD and markers of calcium homeostasis [13, 14]. Although, recent studies have indicated that the measurement of free 25(OH)D may only be useful in certain health conditions that perturb VDBP concentration, such as pregnancy and cirrhosis [35]. A group has recently reported no significant relationship between calculated free 25(OH)D and physical performance (handgrip strength, vertical jump, and aerobic fitness) in 24 Polish football players [27], but this is the only study to date. Therefore, the relevance of reporting free vitamin D metabolites in footballers, particularly in relation to performance outcomes, still requires investigation in studies with much larger sample sizes.

VDBP concentrations were higher at the end of winter than the end of summer and this would, in part, explain the lower calculated free 25(OH)D and free 1, 25(OH)_2_D concentrations at the end of winter. Seasonal variations in VDBP have been reported elsewhere, with increased VDBP in response to low vitamin D status and this may be to maximize uptake of vitamin D from skin [38].

At the end of winter timepoint, there were no differences in PTH, adjusted serum calcium, total 1, 25(OH)_2_D or free 1, 25(OH)_2_D between players who were vitamin D insufficient compared to those who were sufficient. This might be expected as variations in calciotropic hormones in otherwise healthy people may only be seen at extreme total 25(OH)D concentrations. However, calculated free 25(OH)D was higher in those who were sufficient compared to those who were insufficient, as expected due to the strong correlation between the two metabolites [36, 37].

There was no difference in sunlight exposure scores between the two groups but due to the many factors influencing cutaneous synthesis and difficulties in assessing sunlight exposure [20], these findings should be treated with caution. One possible contributor to insufficiency in these players is dietary vitamin D intake. Median intake was significantly lower in the insufficient group (1.6 *μ*g/day) compared to the sufficient group (3.7 *μ*g/day). For both groups, this is lower than the UK Reference Nutrient Intake (10 *μ*g/day) and for the insufficient group this is less than the intake reported by adolescents and younger adults (2.4 *μ*g/day from all sources) in the UK [15]. This may highlight the importance of dietary intake in academy players, especially during the winter period when opportunity for subcutaneous synthesis in the UK is minimal. There was no vitamin D supplementation programme for these players within the academy at the time of study, but it is possible that this might not reflect practices elsewhere.

Our study has several strengths. First, we have used a gold-standard method to assess vitamin D status (first study in UK to report total 25(OH)D using LC-MS/MS in young footballers) and in optimising the timing of assessment to capture the annual peak and nadir for vitamin D status in the UK (end of winter and summer periods). Second, we have also reported calculated free 25(OH)D and 1, 25(OH)_2_D in footballers using a polyclonal assay to measure VDBP. There are limitations of the calculated method for assessing free 25(OH)D and free 1, 25(OH)_2_D, including the assumption of invariant binding affinity of different VDBP alleles with 25(OH)D [39], despite evidence that this is not the case [40]. Future studies in footballers may therefore look to use direct measurement methods for free 25(OH)D. The study has other limitations, including the absence of data corresponding to health and performance outcomes and a relatively small sample size including a loss-to-follow-up of 13 players due to players leaving the football club. There are also known difficulties in quantifying sun exposure due to many influencing factors, including time of day, season, skin type, cloud cover, clothing, and sunscreen use [20]. There are also known difficulties in dietary assessment of vitamin D intakes because it is found in very few food sources. Therefore, consumption/lack of consumption of vitamin D containing foods during the recording period could impact on estimates of habitual intakes.

Future studies need a larger sample size, perhaps across multiple football clubs, and should also consider the relevance of reporting free vitamin D metabolites in footballers, particularly in relation to performance outcomes.

## CONCLUSIONS

We have demonstrated a high prevalence of vitamin D insufficiency at the end of the winter in academy level footballers in the UK, with a fifth still insufficient by the end of the summer. We also observed lower free 25(OH)D at the end of the winter and in those who were insufficient compared to sufficient at the winter timepoint. Vitamin D intake was very low, which suggests a need for targeted dietary advice and appropriate use of supplementation in this cohort of academy level footballers. The high prevalence of deficiency could have implications for performance and injury outcomes (including bone health) in academy players and this should be the focus of further study with consideration given to the relevance of reporting free vitamin D metabolites.
